# Sarcopenic Dysphagia After Occipito-Cervical Fusion Surgery in an Elderly Patient With High-Cervical Myelopathy Caused by Retro-Odontoid Pseudotumor: A Case Report

**DOI:** 10.7759/cureus.11881

**Published:** 2020-12-03

**Authors:** Kousei Miura, Masao Koda, Toru Funayama, Hiroshi Takahashi, Masashi Yamazaki

**Affiliations:** 1 Department of Orthopaedic Surgery, Faculty of Medicine, University of Tsukuba, Tsukuba, JPN

**Keywords:** sarcopenic dysphagia, occipito-cervical fusion surgery, cervical myelopathy, retro-odontoid pseudotumor

## Abstract

Occipito-cervical fusion surgery may cause dysphagia due to inadequate occipito-cervical alignment. However, little is known about any other mechanisms behind postoperative dysphagia. We present a rare case of severe sarcopenic dysphagia despite appropriate occipito-cervical alignment after occipito-cervical fusion surgery. An 85-year-old man who presented with high-cervical myelopathy due to a retro-odontoid pseudotumor underwent occipito-cervical fusion surgery and developed severe dysphagia immediately after the surgery. Swallowing videoendoscopy revealed stagnation of thick fluid at the larynx. Oral intake was prohibited and swallowing rehabilitation was performed. Subsequently, he showed a gradual improvement in swallowing function. He was allowed to start oral intake in the fourth week after surgery and was able to swallow solid foods in the sixth week after surgery. In this case, several parameters of occipito-cervical alignment such as the occipito-C2 angle (O-C2 angle), swallowing line (S-line), C2-C7 angle, and pharyngeal inlet angle, which are recognized as predictors of postoperative dysphagia after occipito-cervical fusion surgery, were adequate to prevent postoperative dysphagia. However, the patient had sarcopenia and cervical hyperlordosis to compensate for thoracic hyperkyphosis, which induces the hypertonicity of hyoid muscles. These findings led to a diagnosis of sarcopenic dysphagia after surgical invasion. Sarcopenic dysphagia is considered to be associated with skeletal and swallowing muscle weakness, apart from thinness, malnutrition, and surgical invasion. Elderly patients with sarcopenia may present with sarcopenic dysphagia because of surgical invasion after occipito-cervical fusion surgery. In such cases, it is important not only to control intraoperative occipito-cervical alignment but also to evaluate preoperative swallowing function.

## Introduction

Occipito-cervical fusion surgery can occasionally cause dysphagia. Revision surgery is needed to improve postoperative dysphagia in some cases. A critical situation may be induced in rare cases with upper airway obstruction in addition to dysphagia. To date, the occipito-C2 angle (O-C2 angle) and the swallowing line (S-line) have been suggested as predictors of postoperative dysphagia after occipito-cervical fusion surgery [1,2]. These predictors can indicate mechanical oropharyngeal stenosis causing dysphagia. However, little is known about other mechanisms behind dysphagia. In this report, we discuss a rare case of severe sarcopenic dysphagia despite appropriate O-C2 angle and S-line after occipito-cervical fusion surgery.

## Case presentation

**Figure 1:**
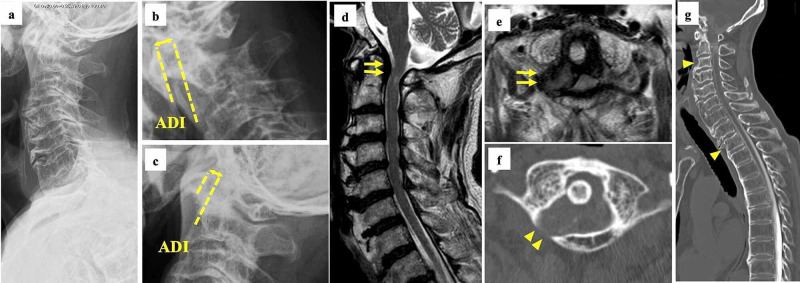
Preoperative images Neutral (a), flexion (b), and extension (c) lateral radiographs showing an increased atlas-dens interval only in the cervical flexion position, which indicates mild reducible atlantoaxial subluxation. Sagittal (d) and axial (e) MRI showing severe compression of the spinal cord at the level of C1 due to a retro-odontoid pseudotumor (arrows). Axial CT (f) revealing C1 posterior arch scalloping due to the pseudotumor (arrowheads). Sagittal CT (g) showing continuous ossification of the anterior longitudinal ligament from C3 to T2 (arrowheads) MRI: magnetic resonance imaging; CT: computed tomography

An 85-year-old man presented with a history of progressive upper limb numbness, loss of manual dexterity, and gait disturbance for several months. The patient had been referred to our hospital for a detailed examination one month before surgery. His height was 160 cm and his body weight was 56 kg [body mass index (BMI): 21.8 kg/m^2^]. His grip strength was 18 kg on the right side and 12 kg on the left side. His skeletal mass index measured using a bioimpedance analysis (BIA) device (MC-780, Tanita, Tokyo, Japan) was 6.9 kg/m^2^. He had difficulty in buttoning his clothes because of the loss of manual dexterity and walked unsteadily. His scapulohumeral reflex was positive and deep tendon reflex in both the upper and lower limb was enhanced. The patient experienced numbness and paresthesia in both his hands. A manual muscle test (MMT) revealed muscular weakness of the left side of the extensor digitorum muscle, flexor digitorum superficialis, the first dorsal interosseous muscle, and abductor digiti minimi (MMT grade 2). He had a mild urinary disorder. The Japanese Orthopaedic Association score for myelopathy was 10 out of 17 points. A plain radiograph revealed an increased atlas-dens interval only in the cervical flexion position, which indicated mild reducible atlantoaxial subluxation. MRI showed severe compression of the spinal cord at the C1 level due to a retro-odontoid pseudotumor. CT revealed C1 posterior arch scalloping due to the pseudotumor, and continuous ossification of the anterior longitudinal ligament (OALL) from C3 to T2. We diagnosed the patient as having high cervical myelopathy due to a retro-odontoid pseudotumor with mild atlantoaxial subluxation (Figure 1).

**Figure 2:**
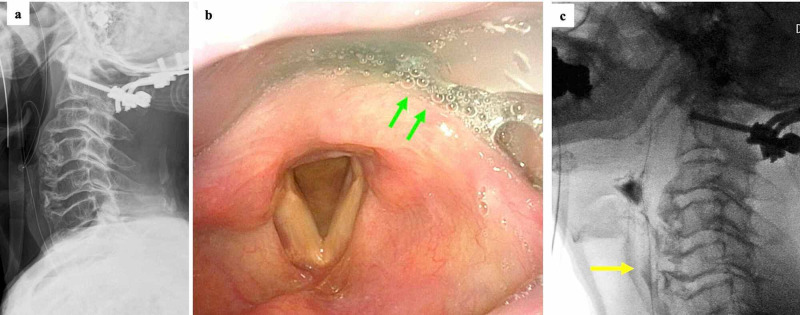
Postoperative images Lateral radiograph (a) after occipito-cervical posterior fusion. Swallowing videoendoscopy (b) on the second day after surgery showing stagnation of thick fluid at the larynx (arrows). Swallowing fluorography (c) in the fourth week after surgery showing improvement of swallowing without stagnation (arrow)

The patient underwent occipito-cervical fusion (O-C2 fusion) and a C1 laminectomy with the use of an occipital plate and a C2 pedicle screw. There were no intraoperative complications. However, the patient complained of severe dysphagia on the day after the surgery despite the absence of preoperative dysphagia. Swallowing videoendoscopy on the second day after surgery found stagnation of thick fluid at the larynx. Oral intake was prohibited and tube feeding was started. His general appearance deteriorated because of aspiration pneumonia and infectious enteritis. However, there was no neurological worsening compared to his preoperative condition. These infectious diseases were improved by antibiotics. A speech therapist started swallowing rehabilitation after the improvement of pneumonia in addition to conventional physical rehabilitation. We performed swallowing fluorography in the fourth week after surgery. The swallowing therapy allowed the patient to swallow without stagnation (Figure 2).

The patient was allowed to start oral intake. His dysphagia gradually improved. Finally, he was able to swallow solid foods and liquid in the sixth week after surgery. His numbness and manual dexterity also improved.

## Discussion

**Figure 3:**
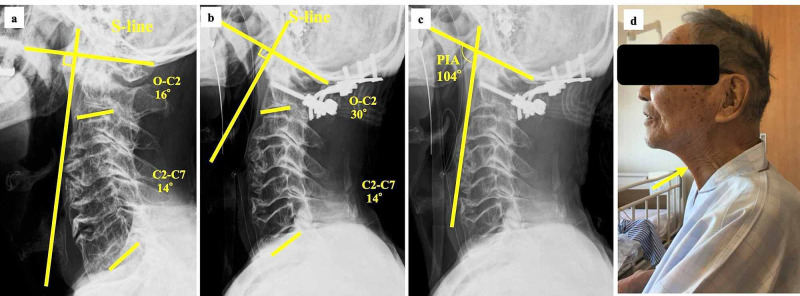
O-C2 angle, S-line, C2-7 angle, and pharyngeal inlet angle Postoperative lateral radiograph (b) showing a larger O-C2 angle, the swallowing line (S-line) at the more anterior position, and no change of the C2–C7 angle compared with the preoperative lateral radiograph (a). Postoperative pharyngeal inlet (PIA) angle measured >90° (c). Postoperative lateral photograph (d) showing hypertonicity of hyoid muscles (arrow) due to cervical hyperlordosis

In this case, transient severe dysphagia occurred immediately after occipito-cervical fusion surgery in an elderly patient with high cervical myelopathy. Izeki et al. [1] have reported that reduction of the O-C2 angle for occipito-cervical fusion caused dysphagia and/or dyspnea due to posterior shift of mandible and oropharyngeal stenosis. Therefore, we made the O-C2 angle larger than the preoperative O-C2 angle at the O-C fusion in the present case. Kaneyama et al. have suggested the S-line (a line passing through the center of the C1 anterior arch and perpendicular to the McGregor line) as a predictor of dysphagia after occipito-spinal fusion surgery [2]. When the apex of cervical lordosis protrudes to the S-line, there is a risk of postoperative dysphagia. In this case, the postoperative S-line was anterior. Regarding the middle-lower cervical alignment, it has been reported that more than a 5° increase of postoperative C2-C7 angle is a risk factor for dysphagia [3]. The postoperative C2-C7 angle did not change and the spinal segments from C2 to C7 were not fused in this case. Direct compression of the pharyngoesophageal segment by OALL is considered to cause dysphagia [4]. A pharyngeal inlet angle of <90° has been suggested as a predictor for dysphagia because of OALL [5]. In this case, the pharyngeal inlet angle was >90° postoperatively. In any case, the dysphagia in this patient was transient. Hence, it was not considered that OALL and postoperative alignment including O-C2 angle, S-line, and C2-C7 angle were the main factors causing dysphagia (Figure 3).

Accordingly, the patient had low grip strength and low skeletal muscle index. It appeared that sarcopenic dysphagia was the main factor for postoperative dysphagia in this patient because this case met the criteria for sarcopenia as defined by the Asian Working Group for Sarcopenia [6]. Sarcopenic dysphagia manifests as difficulty in swallowing due to sarcopenia of generalized skeletal muscles and swallowing muscles [7]. Thinness and skeletal muscle weakness are considered to be associated with dysphagia [8-10]. Moreover, malnutrition and invasions such as surgery are considered to be factors aggravating sarcopenic dysphagia [11]. Love et al. found postoperative dysphagia present in 34% of the patients after hip fracture surgery [12]. Some elderly people, as seen in this case, have cervical hyperlordosis to compensate for thoracic hyperkyphosis. Cervical hyperlordosis induces hypertonicity of hyoid muscles, which results in swallowing dysfunction [13].

The observations described above led us to speculate that the main factor for postoperative dysphagia in the present case was sarcopenic dysphagia due to surgical invasion in addition to hypertonicity of hyoid muscles. Surgical invasions might transiently impair swallowing muscles. However, the possibility that OALL and cervical hypomobility after occipito-cervical fusion surgery were factors for postoperative dysphagia cannot be completely excluded. We recommend evaluating the preoperative swallowing function and paying attention to not only occipito-cervical alignment and OALL but also sarcopenic dysphagia in sarcopenic elderly patients who undergo occipito-cervical fusion surgery.

This case report is in compliance with the Declaration of Helsinki. Written informed consent was obtained from the patient for the publication of this case report and any accompanying images.

## Conclusions

Elderly patients with sarcopenia may present with sarcopenic dysphagia because of surgical invasion after occipito-cervical fusion surgery. It is important not only to control intraoperative occipito-cervical alignment but also to evaluate preoperative swallowing function in such cases.
